# Low temperature-calcined TiO_2_ for visible light assisted decontamination of 4-nitrophenol and hexavalent chromium from wastewater

**DOI:** 10.1038/s41598-019-55912-2

**Published:** 2019-12-18

**Authors:** Mohamed Eid M. Ali, Eman A. Assirey, Shimaa M. Abdel-Moniem, Hanan S. Ibrahim

**Affiliations:** 1 Water Pollution Research Department, National Research Centre, 33 El-Behouth St., Dokki, Cairo, Egypt P.O. 12622,; 2 Chemistry Department, Taibah University, Medinah, Kingdom of Saudi Arabia P.O. 4744,

**Keywords:** Environmental sciences, Environmental social sciences

## Abstract

In the present study, alkaline hydrothermally treated titania nanoparticles (TiO_2_-HT) are prepared and followed by calcination at different low temperatures to improve TiO_2_ activity under visible light. The prepared photocatalysts (PCs) are characterized by different tools. TiO_2_-HT is scrutinized for decontamination of para-nitrophenol (PNP) and hexavalent chromium ions (Cr^6+^ ions) under simulated sunlight. TiO_2_-HT-300 and TiO_2_-HT-400 PCs have nanosized particle with large surface area of 148 and 116.26 m^2^/g, respectively. Additionally, XRD and FTIR proved formation of nanocrystalline anatase TiO_2_. The different calcined TiO_2_-HT materials show lower adsorption capacity for PNP and Cr^6+^ ions. TiO_2_-HT-300 and HT-TiO_2_-400 PCs have higher reduction rate of PNP than that of uncalcined temperature titania (HT-TiO_2_-U) powder. Complete conversion of PNP is achieved at natural pH after 180 min over TiO_2_-HT-300. As well, TiO_2_-HT-300 exhibits a superior photocatalytic removal of Cr^6+^ ions. The enhanced photocatalytic efficacy is ascribed to the synergism between higher surface area and particle size (quantum effect) of TiO_2_-HT-300. As results, HO· radicals are the main key active species for the photocatalytic degradation of PNP over TiO_2_- HT-300 PC but contribution of O_2_^–^ and h^+^ holes is minor. The used method for preparation of TiO_2_-HT-300 reduces the cost preparation as well as environmental impact reduction. Finally, low temperature-calcined TiO_2_ is promising visible light active and an efficient photocatalyst with lower environmental impact for detoxification of PNP and Cr^6+^ ions from water.

## Introduction

As Global warming has represented a severe issue, where the protection of water sources from pollution as well as recycling of industrial effluent is essential responsibility for reducing global warming potentials. Para-nitrophenol (PNP) is extensively applied in the various manufactures; pharmaceuticals, insecticides, fungicides, drugs, and dyes. It was identified in different water streams that received industrial effluents and agricultural run-offs^1–4^. PNP and its derivatives are directly released to the environment with level that formulates risks, due to its non-biodegradability and high toxicity^5^. Upon its toxicity, persistence, and bioaccumulation to humans and animals, PNP is categorized as an emerging contaminant^6^. Human exposure to PNP induces oxygen deficiency and causes various health impacts^7–9^. PNP is considered hazardous organic pollutant. There is a serious need for its degradation from the industrialized and agricultural effluents. The removals of NPs were investigated different remediation techniques^10–18^. Herein, visible light-driven heterogeneous photocatalytic is considered and will be employed for degrading PNP; because photocatalysis technology is an eco-friendly, green and sludge free remediation technique. The lowering of calcination temperature reduces the environmental impact of preparation process via decreasing the electricity consumption. We supposed that the lower size particles (quantum dot effect), high surface area, surface morphology and lowering band gap have important effects on the photocatalytic performance. Previously, the efficiency of TiO_2_ under visible light was chiefly enhanced by ions coupling, existence of different phases, or configuration^19–22^. These trends are costly and complicated. Therefore, the study aimed to improve TiO_2_ via modification of surface area and particle size using facile and simple hydrothermal treatment under alkaline conditions without introduction of metal or non-metal to titania. In this work, TiO_2_ is synthesized using hydrothermal procedure in alkaline media to control surface area and particle size and applied for decontamination of PNP and hexavalent chromium from wastewaters.

## Results and Discussion

### Characterization of photocatalyst

**Figure 1:**
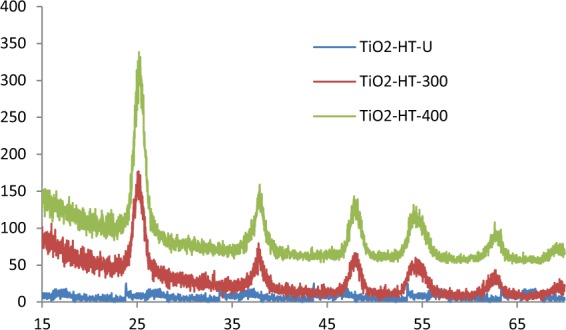
XRD pattern of different prepared photocatalysts.

Powder XRD used to investigate the existence of different crystallographic phase of the prepared materials. The XRD patterns of TiO_2_-HT that prepared by hydrothermal refluxing with alkaline solution followed with calcination at different temperatures (105, 300, 400 °C) are shown in Fig. 1. Hydrothermally treated TiO_2_ sample without post-heating (TiO_2_-HT-U) showed no diffraction peaks indicating amorphous structure of TiO_2_. The diffraction peaks for TiO_2_-HT-300 and TiO_2_-HT-400 are well concurring with those of the standard data (**JCPDS card no. 21-1272**) and has a superior crystalline structure with hexagonal anatase phase and no additional peaks related to rutile phase of TiO_2_ are noticed. Moreover, the calculated d-spacing of 3.53 Å for dominant diffraction line is consistency with the spacing of (101) plane hexagonal anatase phase. The average crystallite size for TiO_2_-HT-300 and TiO_2_-HT-400 was determined by Scherrer equation^23^, it was found to be 8.34 and 10.34 nm, respectively. Furthermore, the reflection width is considerably broadened indicating a small nanocrystalline size which is essential for enhancing the photocatalytic activity of material for degrading the aqueous pollutants.

### FTIR spectra analysis for HT-TiO_2_ PC

FTIR spectra of prepared TiO_2_-HT-300 and TiO_2_-HT-400 materials are analyzed to identify the functional active groups on the surface and showed in Fig. S2. Due to OH stretching vibration, a wide IR band is perceived between 3200 to 3600 cm^−1^ ^24,25^. The wide absorption region below 1000 cm^−1^ is due to the vibration of the Ti-O and Ti-O-Ti bond. The peaks around 1260 and 1430 cm^−1^ in spectra are ascribed to anatase O-Ti-O bonding and Ti-OH and adsorbed water in TiO_2_. These findings confirm the presence of anatase titania.

### N_2_-adsorption/desorption analysis for HT-TiO_2_ PC

**Figure 2:**
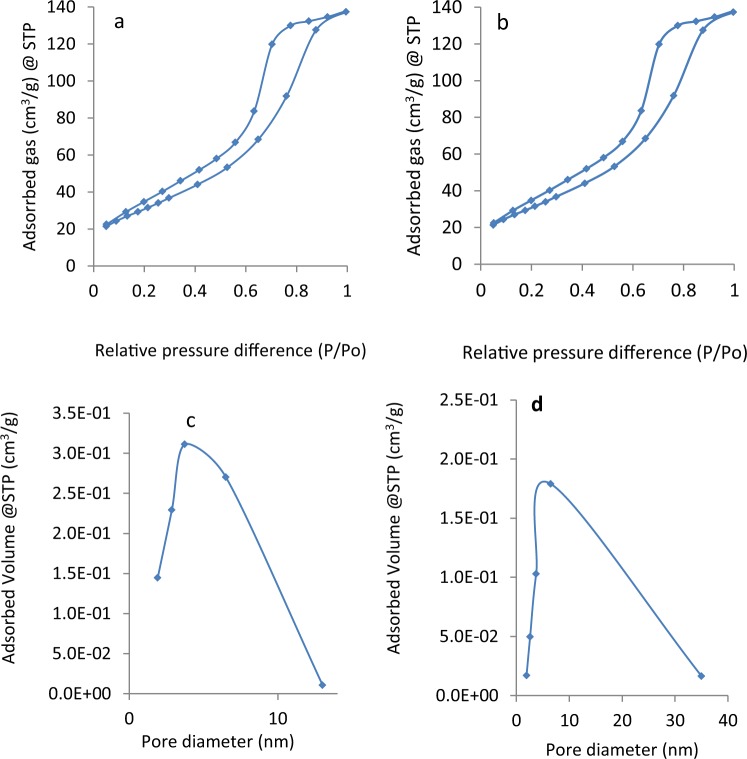
Isotherm of TiO_2_-HT-300 (**a**) and TiO_2_-HT-400 (**b**), pore distribution of TiO_2_-HT-300 (**c**) and TiO_2_-HT-400 (**d**).

Furthermore, N_2_ adsorption/desorption analysis of different prepared HT*-*TiO_2_ is investigated for finding out porosity and surface area and illustrated in Fig. 2. As shown Fig. 2(a,b), adsorption/desorption isotherms for TiO_2_-HT-300 and TiO_2_-HT-400 are similar to IUPAC isotherm III with H3 hysteresis loop in the relative pressure range from 0.2–0.92 indicating mesoporous structure of the prepared material. Figure (2c,d) display the pore size distribution ranges from 1.9 to 13 nm for TiO_2_-HT-300 and TiO_2_-HT-400 samples from 1.95 to 34 nm with corresponding average pore size and pore volume are 3.5 and 6.5 nm, and 0.22 and 0.213 cm^3^/g, respectively. The obtained mesoporosity for TiO_2_-HT-300 and TiO_2_-HT-400 is ought to hydrothermal treatment. The pores TiO_2_-HT-300 and TiO_2_-HT-400 samples ought to condensation of adsorbed water as shown Fig. 2c,d. The specific surface areas are 149 and 116 m^2^/g for TiO_2_-HT-300 and TiO_2_-HT-400, respectively. The higher surface area is due to existence of more pores. The lowering of surface area for TiO_2_-HT-400 is attributed higher temperature of calcination. The superior specific surface area and porous structure of TiO_2_-HT-300 proffer more interfacial sites available for the photocatalytic reaction and thus improve the effectiveness. The photocatalytic activity of prepared materials will be discuss later.

### Scanning electron microscopy (SEM) for TiO_2_-HT PC

SEM is an awfully practical mean for investigation of the surface morphology of TiO_2_ nanoparticle. Figure S3 showed the morphological surfaces of different calcined TiO_2_-HT. Fig. (S3a-b) reveals no existence of definite shape and distribution for TiO_2_-HT-U. But Figures (S3c–e,f-h) depicted homogeneous and uniform distributed particles with nanosized spherical shaped material for TiO_2_-HT-300 and TiO_2_-HT-400, respectively which is consistent with XRD result findings.

### Transmission Electron Microscope (TEM) for TiO_2_-HT-PC

**Figure 3:**
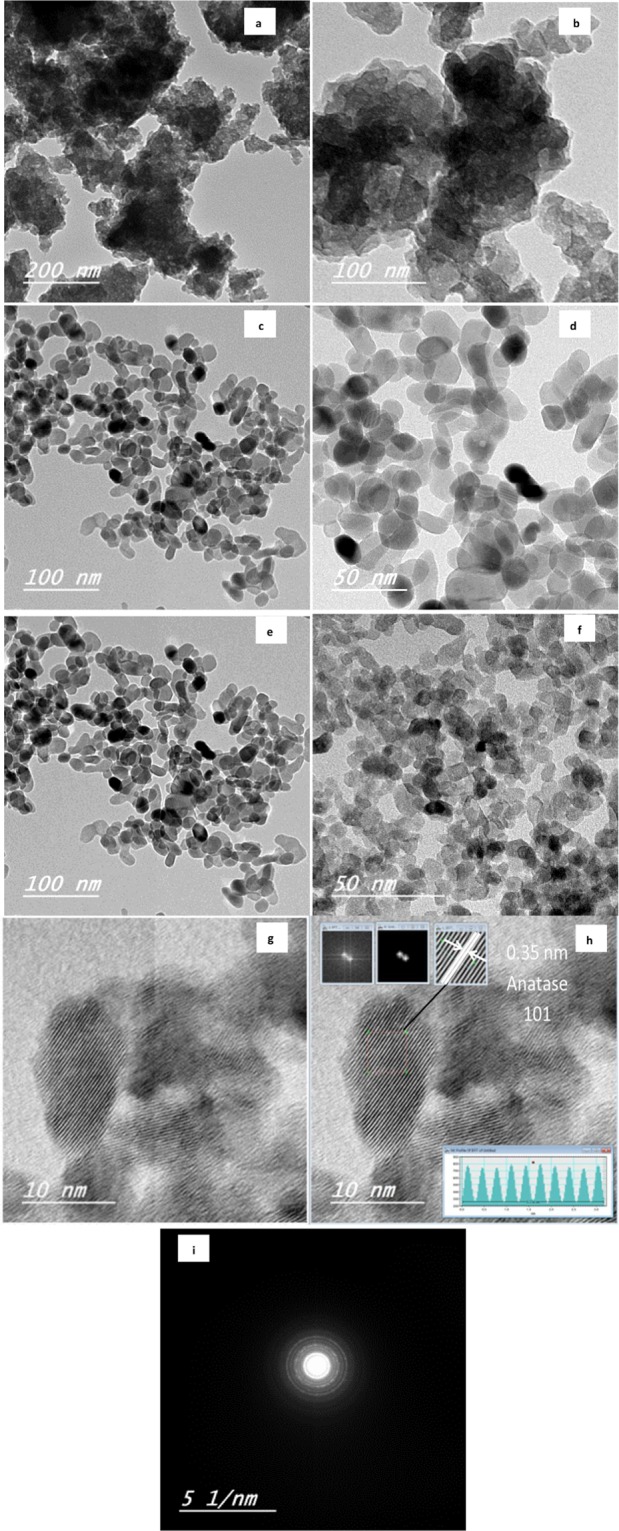
TEM mage of TiO_2_-HT-U (**a,b**), TiO_2_-HT-300 (**c,d,g,h,i**) and TiO_2_-HT-400 (**e**,**f**).

The typical TEM images of TiO_2_-HT with different calcination temperature are depicted in Fig. 3(a–f). Figure 3(a,b) reveals an indefinite crystalline structure for TiO_2_-HT-U, which is consistent with XRD results. Figure 3(c,d,e,f) showed aggregated spherical shape with particle size ranged between 8.3 to 10.4 nm for TiO_2_-HT-300 and 13.5 to 18.4 nm for TiO_2_-HT-400. As well these finding are in consistence with XRD data. High resolution transmission microscopy (HR-TEM) is carried out on higher photoactive materials TiO_2_-HT-300, Fig. 3(g,h) revealed 2D structure TiO_2_-HT-300 with existence definite interplanar spacing of 0.35 nm which ascribed to 101 plane of anatase TiO_2_. These finding somehow demonstrate the consistence with XRD results. Figure 3i showed SEAD and reveals confirming polycrystalline characteristic of TiO_2_PC.

### Particle size distribution for HT-TiO_2_ PC

**Figure 4:**
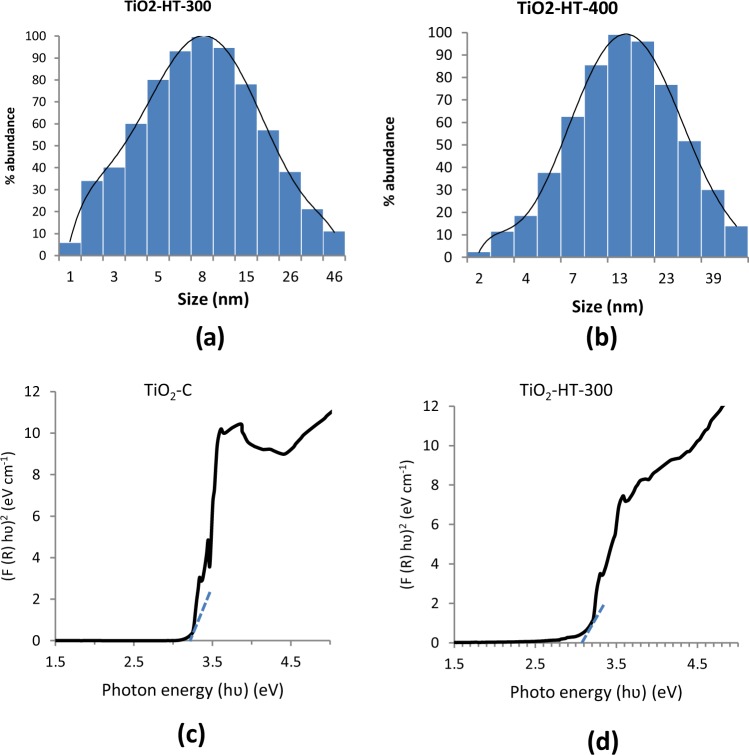
Particle size distribution for TiO_2_-HT-300 (**a**), and TiO_2_-HT-400 (**b**), Optical properties of prepared photocatalysts for TiO_2_-C (**c**), and TiO_2_-HT- 300 (**d**).

Figure 4(a,b) showed the uniform distribution of particle for size TiO_2_-HT-300 and TiO_2_- HT-400. It reveals with particle size between 4 to 20 nm for TiO_2_-HT-300 with average of 8 nm and as well TiO_2_-HT-400 PC shows particles size of 7–30 nm with average size of 13 nm. These findings of particle size are in agreement with size estimation from TEM the XRD analysis for crystalline size.

### Optical properties for HT-TiO_2_ PC

UV–Vis absorption was utilized to estimate the optical characteristics of prepared TiO_2_- HT-300 photocatalysts comparing with commercial TiO_2_ and (Fig. 4c,d). The absorption (A) is shifted to lower energy light (i.e. longer wavelength) for HT-TiO_2_-300 sample than that of commercial titania (TiO_2_-C). Hence, TiO_2_-HT-300 samples have become more active in near visible light region. Kubelka–Munk function was employed to estimate the bandgap energy (Eg) from optical spectra of PC^26^. The relation between hʋ and (hʋ F(R))^1/2^ is depicted for HT-TiO_2_-300 and TiO_2_-C in Fig. 4(c,d). The band gap energies of TiO_2_-C and TiO_2_-HT-300 samples were 3.22 and 3.09 eV, respectively. These results reveal increasing visible light harvesting by TiO_2_-HT-300. The more absorption wavelength range is, the higher the formation rate of electron–hole pairs on the photocatalyst surface increased. Moreover, greatly sequentially more free radicals as well as hydroxyl radicals/holes are produced in solution and improved the degradation activity of PC^27^.

### Photocatalytic activity of prepared photocatalysts

**Figure 5:**
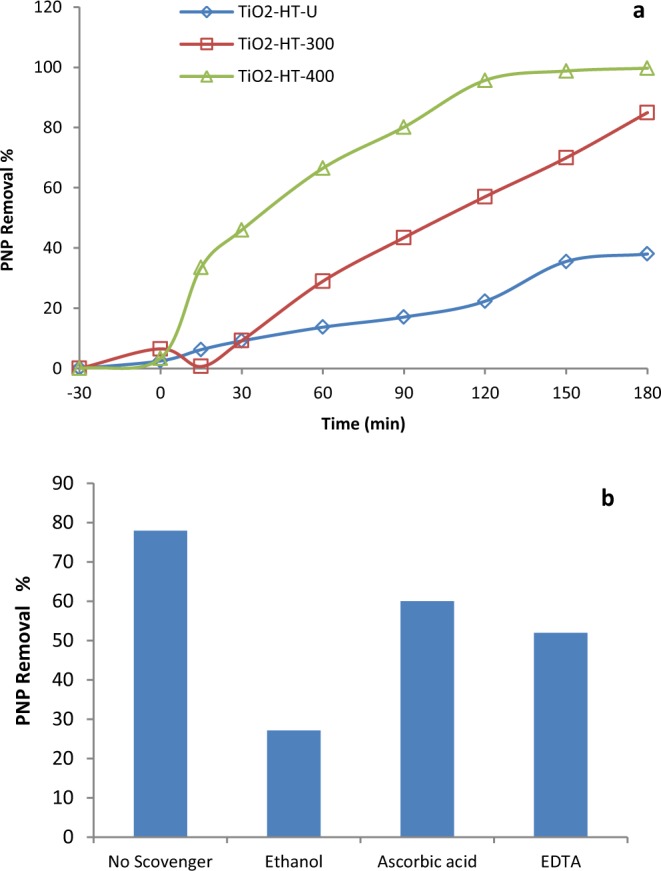
(**a**) Photocatalytic activity of different calcined TiO_2_-HT for photocatalytic removal of 4 NP (Photocatalyst dose 0.5 g/L, PNP concentration 20 mg. L^−1^), (**b**) The photocatalyzed reduction of PNP with different scavenger under visible light irradiation after 90 min over TiO_2_-HT-300.

The photocatalytic removal of PNP (20 mg. L^−1^) over different calcined TiO_2_-HT PC with dose of 0.5 g/L is investigated. Time profile of removal of PNP from aqueous solution is plotted over different calcined TiO_2_-HT under irradiation with visible light (solar simulator) and Fig. 5a. Since, PNP decomposition under visible light irradiation without catalysts was negligible. Meanwhile, the dark adsorption achieved only 3.0–6.3% removal rate over different TiO_2_-HT. It remarkably noticed that TiO_2_-HT-300 and TiO_2_-HT-400 PCs reduced PNP concentration with rate faster than that found for HT-TiO_2_-U powder. Where, only 38% of PNP is reduced after 180 min, it could be owed to uncrystallinity of TiO_2_-HT-U. After 120 min, the superior photocatalytic removal (95%) of PNP is achieved for HT-TiO_2_-300 which higher than that of HT-TiO_2_-400 under visible light (ca. 60%). However, complete reduction of PNP is achieved after 180 min using TiO_2_- HT-300, but only 85% of PNP is removed over TiO_2_-HT-400 under visible light. The results pointed out the importance of calcination treatment of hydrothermally treated TiO_2_. Overall, TiO_2_-HT-300 exhibits a superior activity for degradation of PNP. Consequently, the improved photocatalytic efficacy is endorsed to the synergism of higher surface area and small particle size of TiO_2_-HT-300 with photodegradation activity of photocatalyst^28^. The higher surface area of TiO_2_-HT-300 results in more adsorption and photocatalytic activity compared to TiO_2_-HT-400 and TiO_2_-HT-U. It was revealed that the surface area is supposed as the foremost parameter affecting the photocatalytic activity, where the greater surface area introduces, the more active sites for light harvesting as well as photocatalytic process are, which improves the photocatalytic activity (Abdel Moniem *et al*., 2015; Badawy *et al*., 2015). Moreover, TiO_2_-HT-300 has quantum dot nanoparticle (8.5–10.5 nm) that possibly facilitates the charge separation and prevents recombination of electron–hole pairs^24,25^. As well it is hypothesized that TiO_2_-HT-300 possesses more photocatalytic activity under visible-light, which in consistence with optical properties (See Fig. 4d). Where, the degradation efficiency is enhanced due to decreasing the band gap values of HT-TiO_2_-300 which valorizes the more absorption of visible light leads to more production of free radicals and reactive oxidizing species.

In the current study, TiO_2_-HT-300 PC activity is efficient for PNP removal under solar simulator, that is comparable or higher than that of the previous results. Many PCs were applied for removal of PNP under visible light; TiO_2_, PW12/TiO_2_, 20% g-C_3_N_4_/TiO_2_ and ternary photocatalyst composite of PW12/TiO_2_/g-C_3_N_4_ with dose of 1 g/L and PNP concentration of 20 mg.L^−1^, the conversion of PNP reaches 10%, 20%, 70% and 98.6% under visible-light irradiation, respectively^29^. As well, Ag-AgBr-RGO catalyst is efficient for complete degradation of 5 mg/L of PNP after 180 min of visible light irradiation^30^. Also, over 2 g/L of Bi_2_O_3_ PC, maximum of 100% degradation of PNP within 90 min was achieved meanwhile only 35%, 34% and 22% of PNP was degraded under visible light using TiO_2_, ZnO, and ZrO_2_, respectively within 90 min^31^.

For attainment a good assessment between photocatalytic performances of different hydrothermally treated TiO_2_, the decomposition rate constants (*kapp*) over the samples are computed using pseudo-first-order kinetic model and the data are displayed in Fig. S4. The results revealed that *kapp* for photocatalytic degradation of PNP rate constant are 1.9 × 10^−3^, 7.6 × 10^−3^, and 23.2 × 10^−3^ min^−1^ for TiO_2_-HT-U, TiO_2_-HT-300 and TiO_2_-HT-400 PC, respectively. Obviously, the degradation rate constant of PNP over the TiO_2_-HT-300 and TiO_2_-HT-400 PC is 12.2 and 4 folds higher than that for TiO_2_-HT-U and TiO_2_-HT-400, respectively. It was obviously revealed that TiO_2_-HT-300 has noticeably better photocatalytic efficiency. This improved is confirmed by TEM, optical properties, particle distribution, and BET surface analyses.

Generally, the photoinduced activated species such as entrapped holes (h^+^), hydroxyl radicals (^•^OH) and superoxide radical anions (O_2_^–^) have roles in the photocatalytic process^32^. Moreover, the determining the contributing reactive species in the photocatalytic process is important to investigate the mechanism of the photocatalytic process of PNP degradation. The main active species generated during the photocatalytic destruction process of PNP are scrutinized by entrapping trial. As shown in Fig. 5b, it is found that the removal rates of PNP degradation are reduced to 27, 60 and 52% upon introducing ethanol (•OH radical scavenger), ascorbic acid (O_2_^-•^ radical scavenger) and EDTA (hole scavenger), respectively. Evidently, the introducing ethanol as ^•^OH radicals scavenger, gives a sharp reduction photocatalyzed removal effectiveness to 27%, entail that the free ^•^OH radicals has the major effect on PNP degradation. Meanwhile, in presence of ascorbic acid and EDTA, the photodegrading rate of PNP was slightly decreased, which reveals that the holes and O_2_^-•^ radical are subsidiary contributor in the photocatalytic removal of PNP. Therefore, ^•^OH radicals are main key active species for the photocatalytic degradation of PNP over TiO_2_-HT-300 PC which afford strong indication for the higher photocatalytic ability of TiO_2_-HT-300.

**Figure 6:**
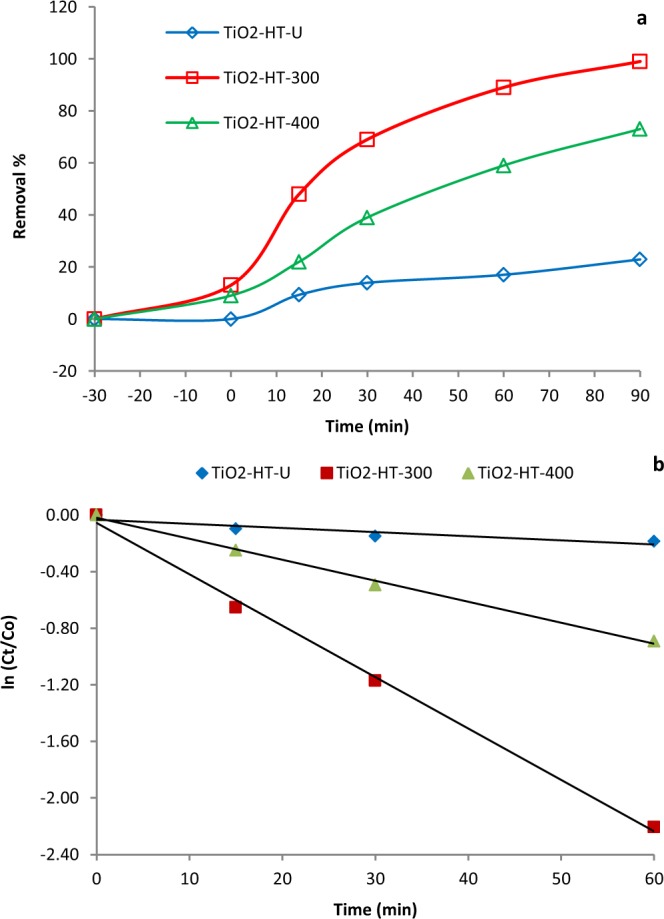
Photocatalytic reduction of Cr^6+^ ion over different PCs under visible light irradiation (**a**), PFO kinetic model (**b**) (Photocatalyst dose 0.5 g/L, Cr^6+^ concentration 20 mg. L^−1^).

The detoxification of 20 mg. L^−1^ of Cr^6+^ from aqueous solution was investigated using TiO_2_-HT-U, TiO_2_-HT-300 and TiO_2_-HT-400; PCs and 60 mg. L^−1^ of formic acid for 180 min under sunlight simulator (UVACUBE 400) and the removal results of Cr^6+^ ions via adsorption and photocatalysis over different TiO_2_-HT. Dark adsorption trials are carried out for 30 min to get adequate adsorption of Cr^6+^ ions on the surface of PCs. It was found that only 13% and 9% of Cr^6+^ ions are removed over TiO_2_-HT-300 and TiO_2_-HT-400, respectively. No adsorption activity is noticed for TiO_2_-HT-U. Control experiment was done without introducing PC in presence of formic acid and no Cr^6+^ ions is remarkable reduced, which revealed that the spontaneous photoreduction of Cr^6+^ ions under visible light is negligible. Figure 6a displayed the photocatalytic reduction curves of Cr^6+^ ions over TiO_2_-HT-U, TiO_2_-HT-300 and TiO_2_-HT-400 PCs. The results showed that Cr^6+^ ions photoreduced by 17%, 89% and 59% after 60 min over TiO_2_-HT-U, TiO_2_-HT-300 and TiO_2_-HT-400 PCs, respectively. Meanwhile, the corresponding noticed photoreduction percentages are 99%, 73% and 23% after 90 min. The photocatalytic reduction efficiency of Cr(VI) ions increases in the order of TiO_2_-HT-300 > TiO_2_-HT-400 > TiO_2_-HT-U after 90 min of simulated sunlight light irradiation. As above mentioned, TiO_2_-HT-300 has the higher proficient activity for photoreduction of Cr^6+^ ions due to separation of photo-generated electron– hole pairs via quantum dots of PC effect, higher surface area and lower bandgap energy (Eg = 3.09 eV). Photoreduction kinetics of Cr^6+^ ions are investigated over different calcined TiO_2_-HT and the time profile of Ln (C/C_0_) illustrated in Fig. 6b. The data showed that *kapps* for photocatalytic degradation of Cr^6+^ ions are 2.9 × 10^−3^, 36.3 × 10^−3^, and 14.8 × 10^−3^ min^−1^ for TiO_2_-HT-U, TiO_2_-HT-300 and TiO_2_-HT-400, respectively. Obviously, the removal rate constant of Cr^6+^ ions over the TiO_2_-HT-300 and TiO_2_-HT-400 PC is 12.5 and 2.5 folds higher than that for TiO_2_-HT-U, TiO_2_-HT-400, respectively.

**Figure 7:**
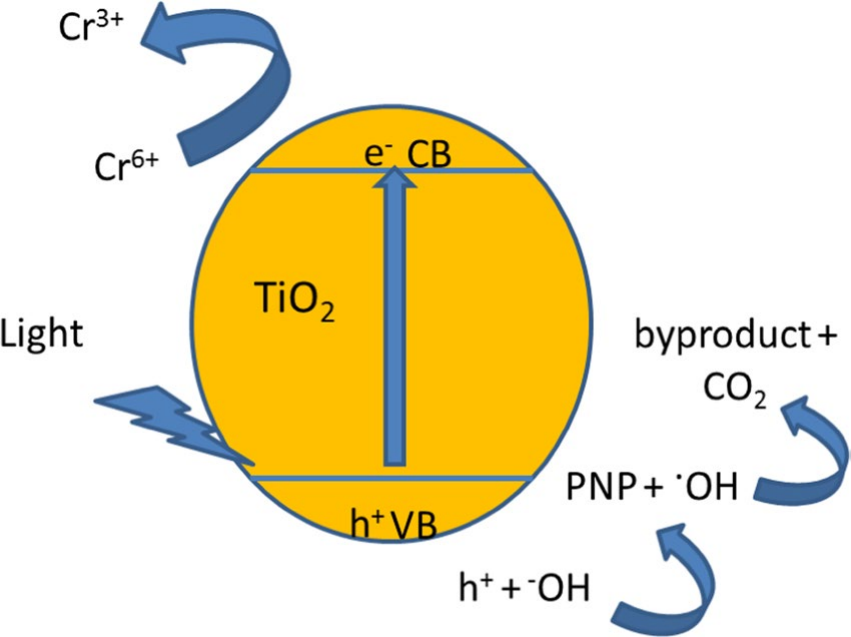
Mechanism of photocatalytic reduction of Cr^6+^ ion and PNP over TiO_2_-HT PC under visible light irradiation.

Based on data (**as shown** Table S1), the photoreduction rate constants (*kapp*) and initial removal rate (r_0_) of Cr^6+^ ions are higher than that of PNP. Upon visible light irradiation of TiO_2_-HT, the adsorbed photon separated electrons-holes pairs (See Fig. 7). Then, electrons transferred to conduction band and shifted toward the surface of PC leading to reduce Cr^6+^ ions directly in case of photoreduction^29^. In case of photocatalytic oxidation, the holes are left in valence band which react with water or hydroxide ions to produce hydroxyl radicals (^•^OH). After that, PNP degraded by ^•^OH attack which will consume more time rather than direct reduction of Cr^6+^ ions. Conclusively, the alkaline hydrothermal treatment for titania with proper calcination temperature possibly improves the photoactivity under sunlight illumination and decontamination of waters from various pollutant; PNP and Cr^6+^ ions. As well, the lower calcined-temperature TiO_2_-HT showed good efficiency rather than the higher calcined-temperature, which results in reduces the energy consumption during the preparation process of materials. Thus, the environmental impact due to electricity use is decreased. Application of TiO_2_-HT-300 is sustainable approach for detoxification of PNP and Cr^6+^ ions from waters.

## Conclusion

Low temperature-calcined titania nanoparticles (TiO_2_-HT) was synthesized for increasing photocatalytic efficiency of TiO_2_ under visible light. The prepared photocatalysts (PC) is characterized by different techniques. HT-TiO_2_ is employed for photocatalyzed decontaminating PNP and Cr^6+^ ions under solar simulator. The obtained results revealed existence of nanosized particle with large surface area. As result, formation nanocrystalline anatase TiO_2_ confirmed XRD and FTIR data. Based on DRS data, hydrothermally treated TiO_2_ become near-visible light photoactive for depollution. Lower fractions of PNP and Cr^6+^ ions are adsorbed over different TiO_2_-HT PCs. TiO_2_-HT-300 and TiO_2_-HT-400 PCs have higher reduction rate of PNP and Cr^6+^ ions under solar simulator. Using solar-driven photoreaction, TiO_2_-HT-300 shows complete removal for PNP and Cr^6+^ ions after 180 min and 90 min, respectively. Photoreduction rate constant of Cr^6+^ ions over TiO_2_-HT-300 is 2.5 and 12.5 times higher than that of TiO_2_-HT-400 and TiO_2_-HT-U, respectively. The synergism of higher surface area and small particle size enhanced photocatalytic efficacy for photodegradation of PNP. As consequences, ^·^OH radicals are major active species for the photocatalytic degradation of PNP over TiO_2_-HT-300. The applied method for TiO_2_-HT-300 preparation decreases the cost preparation and environmental impact reduction due to lowering calcination temperature as well as electricity consumption. As well finally, low temperature-calcined TiO_2_ with solar-driven photoreactor is new approach for detoxification of PNP and Cr^6+^ ions from water/wastewater.

## Experimental

### Preparation of TiO_2_ -HT photocatalyst

Titanium hydroxide gel was synthesized as stated previously^23^, The resulted gel was then dried to make Ti(OH)_4_. To prepare hydrothermally treated titania (TiO_2_-HT), four grams of hp-Ti(OH)_4_ were suspended in 100 mL of 2 M NaOH aqueous solution. This suspension was stirred during 60 min at room temperature and the mixture was transferred into round flask, and then was refluxed for 24 h at 160 °C. The obtained material was washed with H_2_O and filtered off. This washed material was suspended in 500 mL of aqueous solution of HCl (0.1 N) and stirred for 24 h. The treatment with HCl was repeated 3 times in order to remove the residual sodium ions. Then, the materials were washed with deionized water several times to remove chloride. These formed materials were dried in oven at 105 °C. Finally, the prepared powders were calcined at different temperatures. The prepared catalysts were denoted with TiO_2_ HT–U, TiO_2_- HT-300 and TiO_2_-HT-400 for catalyst and treated at temperature of 105, 300 and 400 °C, respectively.

### Characterization of HT-TiO_2_ PC

The characterization of materials are previously described^24,26,28^, powder X-ray diffraction (XRD) was performed on a PANalytical X’PertPRO X-ray diffractometer using filtered Cu Kα radiation (λ = 0.154 nm). Fourier transform infrared (FTIR) spectroscopy was conducted by using a BRUKER VERTEX 70. Transmission electron microscopy (TEM) images were taken using a JEOL JSM-1200 EX II operating at 100 kV. Scanning electron microscopy (SEM) images were taken using a JEOL JSM-7600F field emission scanning electron microscope. High-resolution transmission electron microscopy (HRTEM) images were taken with a Tecnai 20 G2S-Twin operating at 200 kV. Nitrogen adsorption–desorption isotherms were measured at 77 K with a Micromeritics ASAP 2010. The specific surface areas were calculated by the Brunauer–Emmett–Teller (BET) method. Zeta-potential analysis was performed on a Malvern Zetasizer Nano ZS. DSR spectra were obtained in air at ca. 300 K in the wavelength range 200–900 nm using a Shimadzu UV-2401 PC spectrophotometer with BaSO_4_ as the reference material.

### Photocatalytic Activity for HT-TiO_2_ PC

A Pyrex batch reactor of beaker with volume of 250 mL, was used for performing the reactivity experiments. Illumination is provided by solar simulator UVACUBE 400, Honle, Germany. The photocatalytic activity of prepared photocatalyst is tested by reactions of photo-degradation of 4-nitrophenol. 100 ml of 20 ppm of pollutant (PNP, Cr^6+^ ions) was added to photoreactor. The photocatalyst of 50 mg was suspended in 100 ml of aqueous solution of pollutant and then stirred for 30 min to make the photocatalyst homogeneously dispersed into the solution of reaction. Then, the solar simulator was turned on, sample were taken at time interval. The concentration of 4-nitrophenol and Cr^6+^ ions are analyzed by a JASCO- V-730, UV–Visible recording spectrophotometer at λ_max_ = 318 and 350 nm, respectively. Before determination, the withdrawn suspensions are filtered with syringe filter (Whatman, 0.45 µm). The pathway of photocatalytic mechanism is investigated in presence of ethanol, ascorbic acid (AA), and ethylene diamine tetra acetate (EDTA) with concentration (2 mM) for each one as ^•^OH radical, O_2_^−•^ radical and holes scavengers, respectively.

## Supplementary information

Supplementary information.
