# Supplementary material for: Low temperature-calcined TiO2 for visible light assisted decontamination of 4-nitrophenol and hexavalent chromium from wastewater
Source: Sci Rep. 2019 Dec 18;9:19354. doi: 10.1038/s41598-019-55912-2 (PMC6920423; doi:10.1038/s41598-019-55912-2)
Supplement: Supplementary file 1 — Supplementary information. [file 41598_2019_55912_MOESM1_ESM.pdf]

Supplementary information

Low temperature-calcined TiO_2 for visible light assisted decontamination of 4-nitrophenol and hexavalent chromium from wastewater

Mohamed Eid M. Ali[1,*], Eman A. Assirey[2,], Shimaa M. Abdel-Monein[1], Hanan S. Ibrahim[1]**

[1]Water Pollution Research Department, National Research Centre, El-Buhouth St., Dokki, Cairo, Egypt, P.O. 12622.

[2]Chemistry Department, Taibah University, Medinah, Kingdom of Saudi Arabia, P.O. 4744

*Corresponding Author: Tel: +201008457583, email: alienv81@yahoo.com

**Corresponding Author: Tel: +966553311211, email: eman_assirey@hotmail.com

Material and methods

Chemical used

Chemicals used in the research work are graded for analytical used (titanium chloride, ethanol, para-nitrophenol (Fig.S1) sodium hydroxide, hydrochloric acid 37 %), potassium dichromate as source of hexavalent chromium ions and deionized water is used in preparation and treatment study.

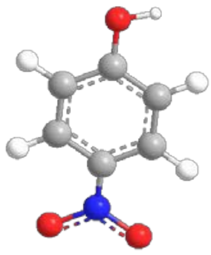

Grey : Carbon, White,: Hydrogen, Blue: Nitrogen, Red: Oxygen Figure

S1 structure of Para-nitrophenol (PNP)

Results and Discussion

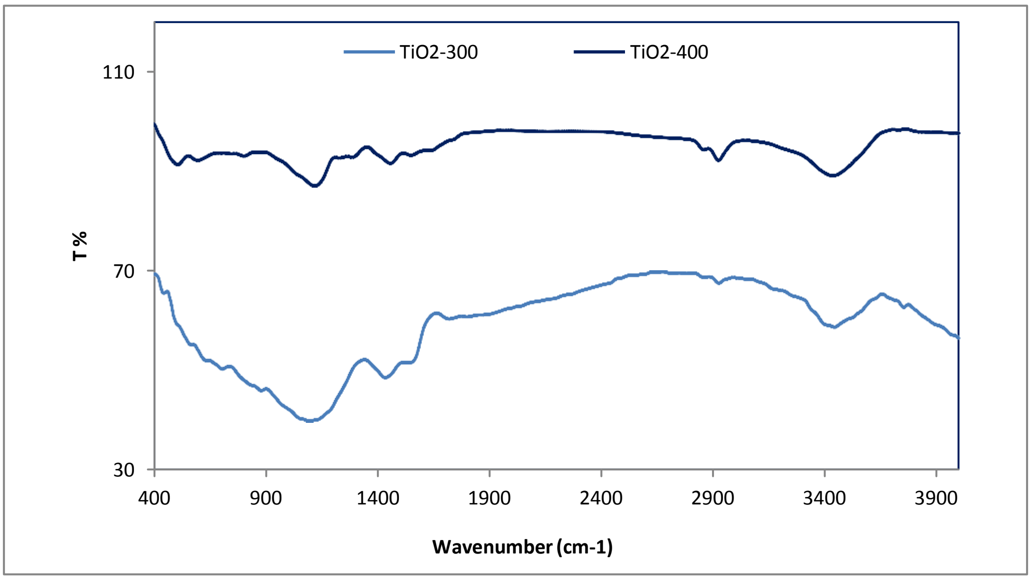

Fig. S2 FTIR of prepared HT-TiO_2-300 and HT-TiO_2-400 materials

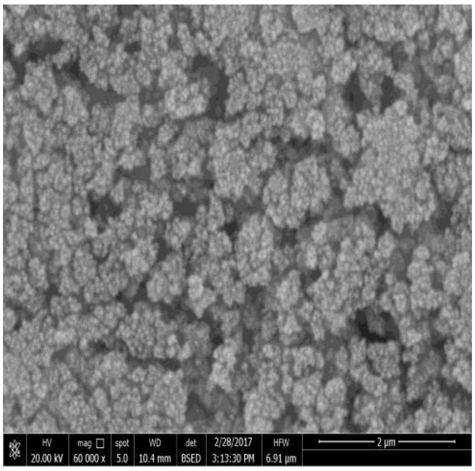
HV 20.00 kV
mag 60 000 x
spot 5.0
WD 10.4 mm
det BSED
2/28/2017 3:13:30 PM
HFW 6.91 µm
2 µm

a

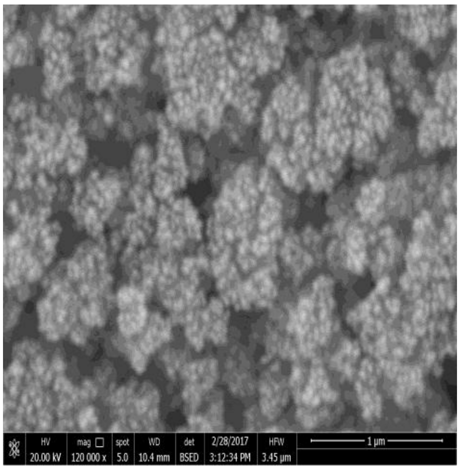
HV 20.00 kV
mag 120 000 x
spot 5.0
WD 10.4 mm
det BSED
2/28/2017 3:12:34 PM
HFW 3.45 µm
1 µm

b

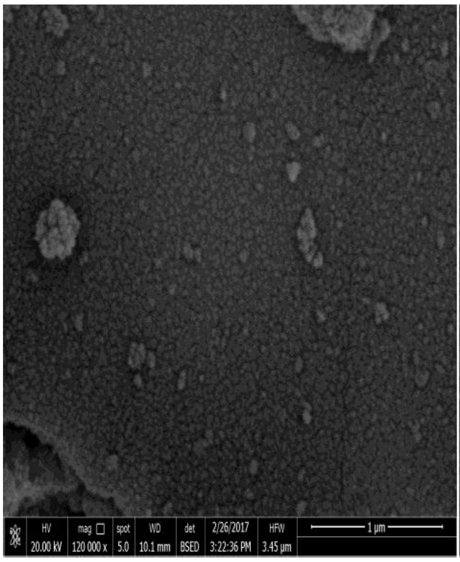
HV 20.00 kV
mag 120 000 x
spot 5.0
WD 10.1 mm
det BSED
2/26/2017 3:22:36 PM
HFW 3.45 µm
1 µm

c

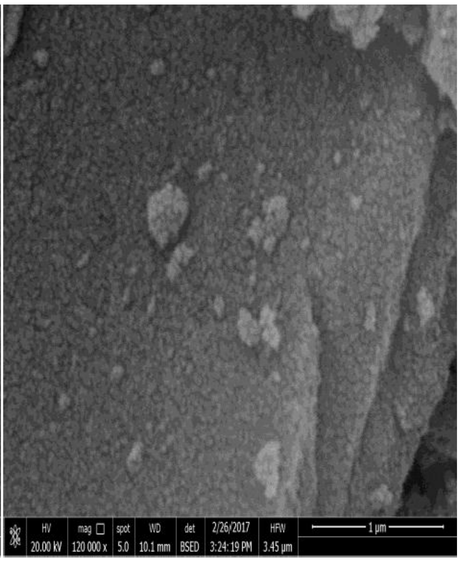
HV 20.00 kV
mag 120 000 x
spot 5.0
WD 10.1 mm
det BSED
2/26/2017 3:24:19 PM
HFW 3.45 µm
1 µm

d

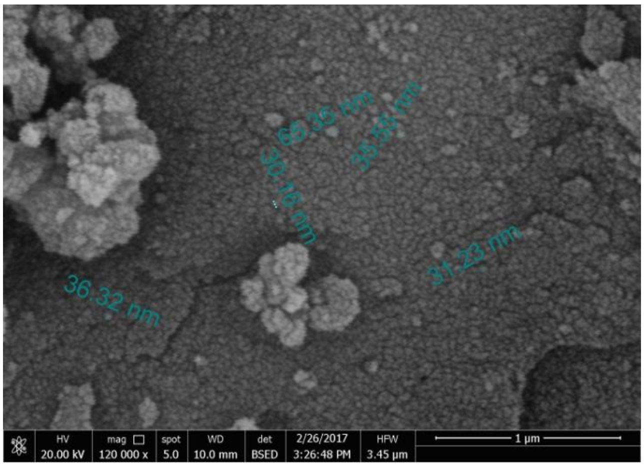
HV 20.00 kV
mag 120 000 x
spot 5.0
WD 10.0 mm
det BSED
2/26/2017 3:26:48 PM
HFW 3.45 µm
1 µm
36.32 nm
31.23 nm

e

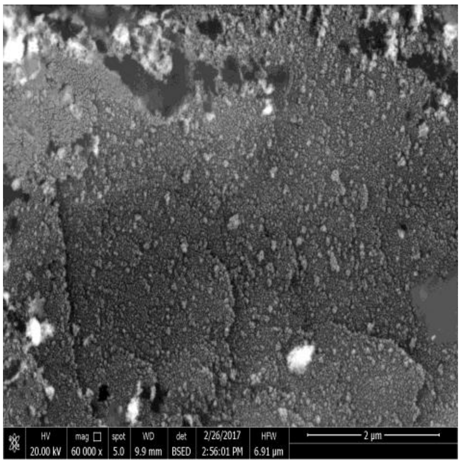

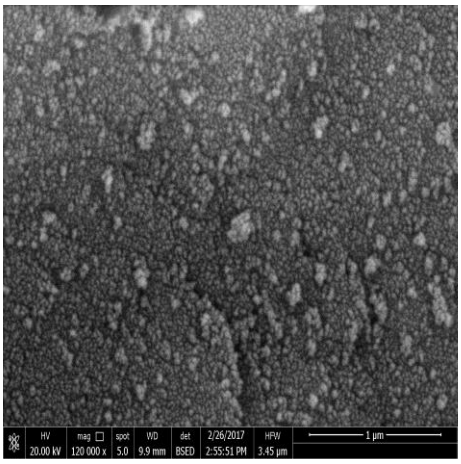

f **g**

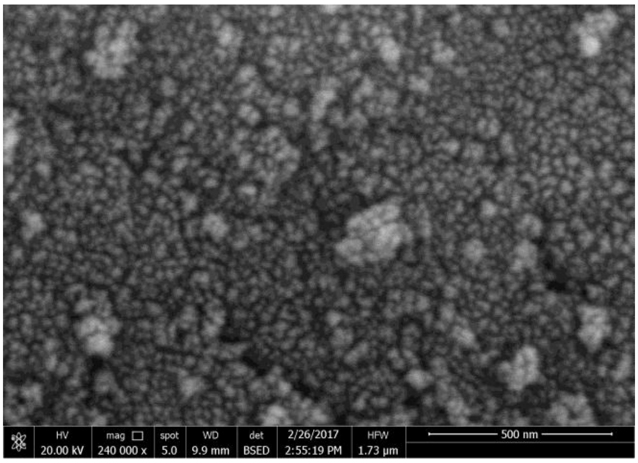

h

Fig. S3 SEM image of prepared photocatalysts; HT-TiO_2-U (a-b), HT-TiO_2-300 (c-e) and HT-TiO_2-400 (f-h)

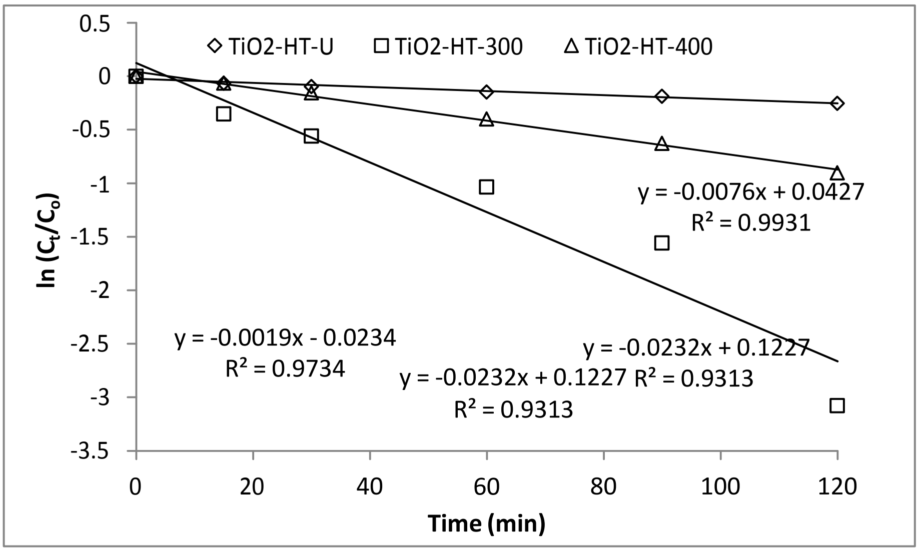

Fig S4 first order kinetic model for degradation of PNP

Table S1 First order kinetic models for photocatalytic reduction of PNP and Cr^{6+} ions using different calcined TiO_2-HT

PC	*kapp* (min^{-1}) x 10^{-3}		r_0 x 10^{-3} (mg. L^{-1}. min^{-1})	
	Cr^{6+} ions	PNP	Cr^{6+} ions	PNP
TiO_2-HT-U	2.9	1.9	58	38
TiO_2-HT-300	36.4	23.2	756	464
TiO_2-HT-400	14.8	7.6	296	152
